# The role of inflammatory factors and T‐cell subsets in the diagnosis of recurrence in epithelial ovarian cancer patients and the effect of olaparin treatment on them

**DOI:** 10.1002/iid3.1059

**Published:** 2023-10-25

**Authors:** Gulijianati Maowulieti, Shaojie Zhao, Min Zhao, Hua Yuan

**Affiliations:** ^1^ Department of Gynaecology, Wuxi Maternity and Child Health Care Hospital Women's Hospital of Jiangnan University, Jiangnan University Wuxi Jiangsu China

**Keywords:** epithelial ovarian cancer, inflammatory factor, olaparib, T‐lymphocyte subsets

## Abstract

**Background:**

The aim of the study is to investigate the role of serum inflammatory factors and T‐cell subsets in the diagnosis of recurrence in epithelial ovarian cancer patients and the effect of olaparib on inflammatory factor and T‐lymphocyte subsets in patients with recurrent epithelial ovarian cancer.

**Methods:**

In this study, 100 patients diagnosed as recurrent epithelial ovarian cancer in our hospital and 100 patients without recurrent epithelial ovarian cancer in the same period were selected. According to the treatment plan, the recurrent patients were divided into conventional therapy group (Paclitaxel and Carboplatin) and combined therapy group (Paclitaxel, Carboplatin, and olaparib). The levels of serum inflammatory factors were evaluated by enzyme‐linked immunosorbent assay. The peripheral blood T‐lymphocyte subsets in each group were detected by flow cytometry.

**Results:**

Compared with nonrecurrent patients, recurrent patients have higher serum interleukin‐6 (IL‐6) and tumor necrosis factor‐α (TNF‐α) levels (*p* < .05), and lower interferon‐γ (IFN‐γ) level and the CD4+/CD8+ ratio. After adjusting for confounding factors, the results showed that the serum IL‐6, IFN‐γ, and TNF‐α levels were influencing factors of recurrence in epithelial ovarian cancer patients. The area under the receiver operating curve and the sensitivity of serum TNF‐α in predicting ovarian cancer recurrence were higher than those of IL‐6 and IFN‐γ. After secondary chemotherapy and/or olaparib maintenance treatment, the IL‐6 (*p* < .001) and TNF‐α (*p* < .001) levels in combined therapy group were lower than those in the conventional therapy, whereas the IFN‐γ level (*p* < .001), the CD4+ T‐cell proportion (*p* = .0069) and the CD4+/CD8+ ratio (*p* = .0201) were higher than those in the conventional therapy.

**Conclusion:**

The serum IL‐6, TNF‐α, and IFN‐γ levels were closely related to the recurrence of ovarian cancer. Olaparib maintenance treatment can significantly decrease the IL‐6 and TNF‐α level, and increase IFN‐γ level and the CD4+/CD8+ ratio in patients with recurrent ovarian cancer.

## Graphical abstract

This study indicate that olaparib can significantly improve the immune function of patients with recurrent ovarian cancer.

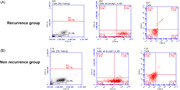

## 1 INTRODUCTION

Ovarian cancer is the second most common cause of gynecologic cancer death in women around the world with a 5‐year survival rate of ~47%.^1^ With the development of technology, the diagnosis and treatment level of ovarian cancer patients has significantly improved. However, most patients are diagnosed at a late stage and survival rate remains dismal.^2^, ^3^ In recent decades, despite significant progress in surgical techniques such as laparoscopy and robotic systems, as well as chemotherapy, have made significant progress, the recurrence rate is still as high as 70%–80%.^4^ Therefore, the prevention and treatment of ovarian cancer recurrence is an urgent problem to be solved.

The maintenance treatment of ovarian cancer is highly regarded. In fact, as early as the late 1980s, the term maintenance therapy appeared in the literature of ovarian cancer. Initially, the researchers tried intravenous interferon‐γ (IFN‐γ) as a maintenance treatment for recurrent ovarian cancer. Subsequently, the national comprehensive cancer network ovarian cancer clinical practice guide once recommended paclitaxel or pazopanib as the drug choice for maintenance treatment of ovarian cancer. However, maintenance treatment has not been really implemented in clinic for a long time, and maintenance treatment is not encouraged for patients with ovarian cancer, mainly because of serious drug toxicity and side effects or little survival benefit. Until the emergence of anti angiogenesis drugs and molecular targeted drugs such as poly‐adenosine diphosphate ribose polymerase inhibitor (PARPi), the current situation of maintenance treatment of ovarian cancer has been really improved.^5^, ^6^ The establishment of maintenance therapy for ovarian cancer has changed the previous treatment mode and strategy of ovarian cancer, and provided new hope for improving the poor prognosis of ovarian cancer.

PARPi, represented by olaparib, nilapaparib, and lucaparib, is a very popular molecular targeted drug in the field of ovarian cancer research in recent years. It has become a new choice for maintenance treatment of ovarian cancer patients.^7^ They mainly inhibit the repair of DNA single‐strand damage in cancer cells, resulting in the continuous accumulation of DNA single‐strand damage, resulting in DNA double‐strand breaks. In cancer cells with DNA homologous recombination defect, double‐strand breaks cannot be repaired with high fidelity, resulting in cytotoxicity and “synthetic lethal effect.”^8^ A randomized study^9^, ^10^ confirmed the excellent efficacy of olaparib in second‐line maintenance therapy in platinum‐sensitive recurrent ovarian cancer patients. Subsequently, the researchers applied olaparib to the first‐line maintenance treatment of newly diagnosed ovarian cancer and achieved very encouraging research results.^11^

In the normal body, each T‐lymphocyte subgroup interacts and maintains the normal immune function of the body.^12^ According to the cell surface markers, they were divided into different functional subgroups by monoclonal antibodies. Once the number of lymphocyte subpopulations is abnormal, the human body experiences immune disorders and a series of pathological changes.^13^ Mature T lymphocytes can express CD3 molecules on their surface, whereas CD4 and CD8 cannot be simultaneously expressed on the surface of mature T lymphocytes. Therefore, mature T lymphocytes can be divided into two subgroups: CD4^+^ T cells and CD8^+^ T cells.^14^ The detection of T‐lymphocyte subsets in the blood is an important method to observe the cellular immune level of the body, which plays an important role in the diagnosis, treatment, and prognosis of malignant tumors,^15^ autoimmune diseases^16^ and immune deficiency disease.^17^ At present, more and more studies show that T‐lymphocyte subsets have abnormal changes in ovarian cancer^18^ and different T lymphocytes have an important impact on the ovarian cancer development.^19^ The decrease of CD4^+^/CD8^+^ ratio is an important feature of immune deficiency disease.^20^ Serum human epididymis secretory protein‐4 (HE4) is one of the most sensitive tumor markers of ovarian cancer found in recent years. Its serum level can be used as a good index to monitor the recurrence of ovarian cancer patients after chemotherapy.^21^ The role of interleukin‐6 (IL‐6), tumor necrosis factor‐α (TNF‐α), and IFN‐γ in the development of ovarian cancer has attracted much attention.^22^

Therefore, this study compared the proportion of serum cytokines and T‐lymphocyte subsets in recurrent and nonrecurrent epithelial ovarian cancer patients. Subsequently, recurrent ovarian cancer patients received two different treatments (routine treatment or routine treatment combined with maintenance treatment). The proportion of serum cytokines and T‐lymphocyte subsets in the two groups were compared after treatment to explore the application value of olaparib maintenance treatment in the treatment of recurrent ovarian cancer.

## 2 MATERIAL AND METHODS

### 2.1 Research object

Retrospective analysis of patients with epithelial ovarian cancer admitted to Wuxi maternal and Child Health Hospital from January 2019 to December 2020. After undergoing tumor cell reduction surgery, the serum levels of CA125 and HE4 in all patients decreased to normal (CA125 ≤ 35 U/mL, HE4 ≤ 72 pmol/L). After complete remission of clinical symptoms, patients were followed up at the outpatient clinic every 3 months for 1 year. Based on imaging and pathology, the recurrence of epithelial ovarian cancer was determined. Within the 1‐year follow‐up period of patients, patients with recurrence will be included in the recurrence group, and those who did not relapse were included in the nonrecurrent group. In this study, patients with recurrent ovarian cancer (*n* = 100), who met the diagnostic criteria were selected, and 100 patients without recurrent ovarian cancer in the same period were selected.

Inclusion criteria were as follows: (1) the patient was diagnosed with ovarian epithelial carcinoma through clinical, imaging, and pathological tests; (2) Federation International of Gynecology and Obstetrics (FIGO) is divided into Stages III or IV; (3) the patient's age is between 18 and 70 years old; (4) the patient receives chemotherapy treatment for this disease for the first time; (5) all patients underwent tumor cell reduction surgery; and (6) the patient has no other malignant tumors. Exclusion criteria were as follows: (1) patients cannot tolerate chemotherapy treatment; (2) patients with primary organ dysfunction, coagulation dysfunction, and other malignant tumors; (3) patients with severe infection, pulmonary disease, uncontrolled blood sugar of diabetes, untreated severe hypertension, congestive heart failure, arrhythmia, and cardiac infarction; (4) patients with peripheral neuropathy; (5) patients with mental disorders or patients with poor compliance due to other reasons; (6) patients with a history of chemotherapy treatment; (7) patients with an expected survival period of <6 months; (8) pregnant or lactating women; (9) those who are allergic to any drug allergy of carboplatin or paclitaxel.

### 2.2 Grouping patients for treatment

According to the treatment plan, the recurrent patients were divided into conventional therapy group (*n* = 50) and combined therapy group (*n* = 50). All patients were treated with conventional paclitaxel combined with carboplatin. Three weeks are a course of treatment, a total of six courses of chemotherapy. Based on conventional therapy (Paclitaxel and Carboplatin), the combined therapy group was treated with olaparib to maintenance therapy. This study was approved by the ethics committee of Wuxi maternal and Child Health Hospital (No. 2020‐01‐0731‐26).

#### 2.2.1 Conventional therapy

The paclitaxel (H20066640) is produced by Beijing Shuanglu Pharmaceutical Co., Ltd. The carboplatin (H20020181) is produced by Qilu Pharmaceutical Co., Ltd. On the first day, the patient received intravenous infusion of 24 h of paclitaxel injection (135 mg/m^2^), 20 mg of dexamethasone 12  and 6 h before intravenous infusion of paclitaxel, 25 mg of Promethazine intramuscular injection 30 min before intravenous infusion, and injection carboplatin (area under the receiver operating curve [AUC] = 5) + 5% 500 mL of glucose on the second day.

#### 2.2.2 Combined therapy

Three weeks are a course of treatment, and inject paclitaxel (135 mg/m^2^) and carboplatin (AUC = 5) intravenously on the first day of each course. After six courses of chemotherapy, patients take Olapanide orally (twice a day, 300 mg each time) until the disease progresses. During the chemotherapy period, the patient's vital signs and clinical indicators were closely monitored, electrocardiographic monitoring was performed, and 24 h urine output was recorded. And provide prevention and treatment such as diuresis, antiemesis, and stomach protection. If white blood cells are less than 3.0 × 10^9^/L during treatment or after therapy, then inject 150 ~ 300 μg of recombinant human granulocyte stimulating factor subcutaneously.

### 2.3 Measurement of TNF‐α, IL‐6, and IFN‐γ levels by enzyme‐linked immunosorbent assay (ELISA) kits

All subjects were collected 3–5 mL of venous blood on an empty stomach after treatment. The collected blood was allowed to stand at room temperature for 30 min, centrifuged at 3000 rpm for 15 min. Then the serum was collected and stored at −80°C for standby. According to the manufacturer's instructions, the serum TNF‐α, IL‐6, and IFN‐γ levels were determined by TNF‐α (ab46087, Abcam), IL‐6 (ab46042), and IFN‐γ (ab46048) ELISA kits.

### 2.4 Detection of T‐lymphocyte subsets in peripheral blood

The red blood cell lysate was added into the blood sample according to the volume of 1:5, to lyse at room temperature for 15 min. The sample was centrifuged at 1000 rpm for 5 min. The precipitates were resuspended and centrifuged. After washing the precipitates with phosphate‐buffered saline three times, white precipitates are lymphocytes. Then, the lymphocyte precipitation was resuspended with buffer containing 2% fetal bovine serum, and divided into two parts. One part is added with negative control antibody and the other part is added with 5 μL FITC‐CD4 antibodies (Biolegend cat 300505), APC‐CD3 antibodies (Biolegend cat 300311, and PE‐CD8 antibodies (Biolegend cat 344705). The cells were gently mixed, incubated, and then detected by flow cytometry using BD FACSVerse^TM^ System.

### 2.5 Statistical analysis

Expressed as mean ± SD, analyzed by the SPSS 25.0 (IBM) statistical software, and comparisons between two groups were performed using the Student's *t* test. Counting data are expressed in *n* (%) and comparisons between two groups were performed using *χ* ^2^ test. The value of serum inflammatory factors in the diagnosis of ovarian cancer recurrence using receiver operating curve (ROC) analysis, and the factors influencing ovarian cancer recurrence using logistic regression analysis, with *p* < .05 represents a statistically significant difference.

## 3 RESULTS

### 3.1 Comparison of general information between two groups of patients

A total of 100 patients with recurrent ovarian cancer and 100 patients without recurrence in the same period were collected. Our results showed significant differences in age, FIGO, and differentiation grade between the nonrecurrent and recurrent groups of patients (Table 1).

**Table 1:** Comparison of general information between two groups of patients.

Group		Recurrent (*n* = 100)	Nonrecurrent (*n* = 100)	*χ* ^2^	*p*
Age	≥50	24	76	30.943	.000
	<50	63	37		
FIGO	III	35	65	11.562	.001
	IV	59	41		
Differentiation Grade	Low	39	61	5.138	.023
	Medium/high	55	45		

### 3.2 Comparison of inflammatory factors and T‐lymphocyte levels between nonrecurrence group and recurrence group

**Figure 1:**
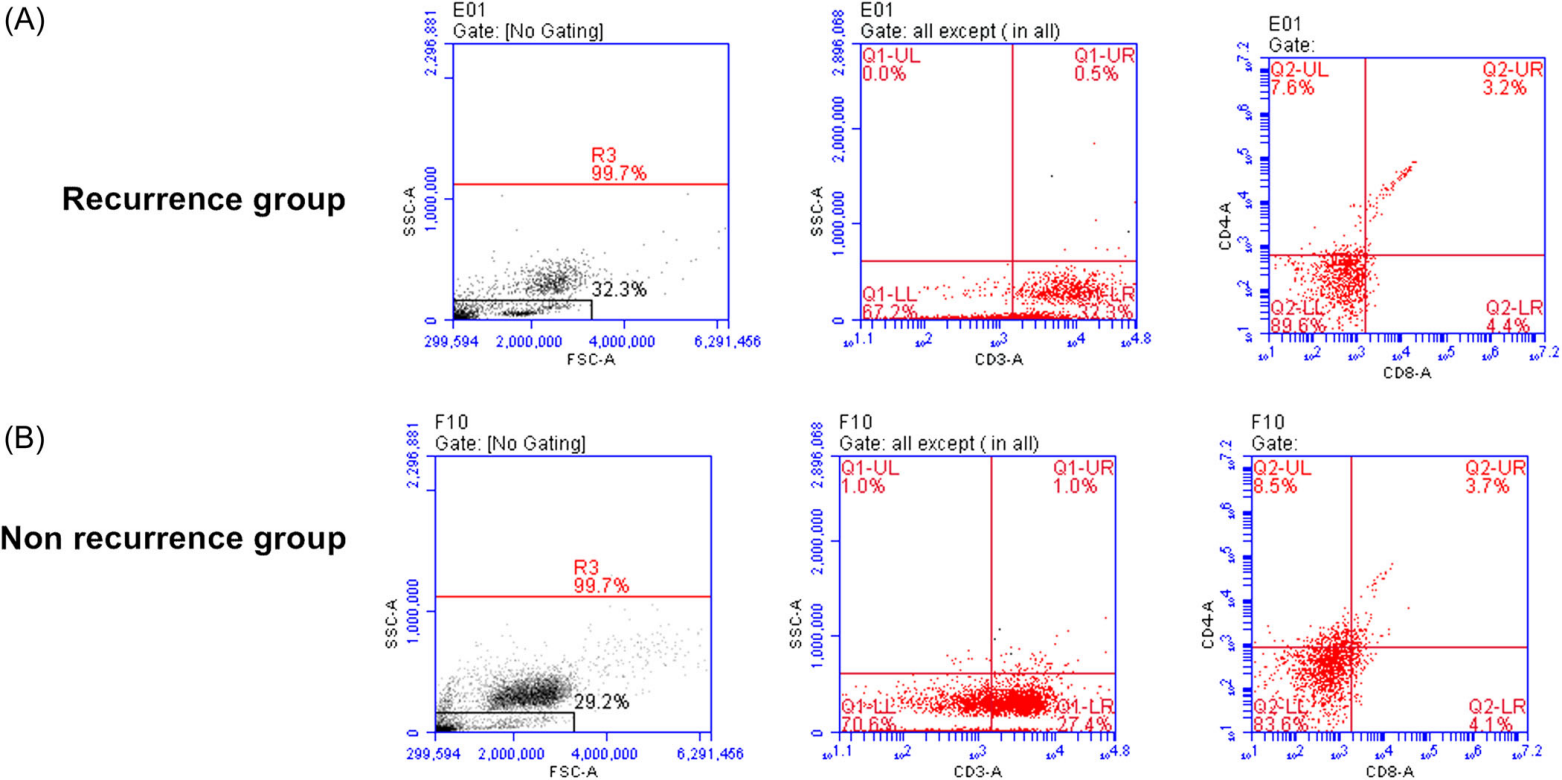
The proportion of CD3^+^ T (C), CD8^+^ T (D), CD4^+^ T (E) cells and CD4^+^/CD8^+^ ratio (F) in peripheral blood of patients in two groups. The proportion of T‐cell subsets in peripheral blood of patients in nonrecurrence group (A) and recurrence group (B) were detected by flow cytometry.

As shown in Table 2, the levels of serum IL‐6 and TNF‐α in the recurrence group were significantly higher than that in the non‐recurrence group, whereas the IFN‐γ level was significantly lower. The peripheral blood CD3^+^, CD4^+^, CD8^+^ T‐lymphocyte levels between the two groups were detected by flow cytometry (Figure 1). There was no significant difference in the CD3^+^ T and CD8^+^ T‐cell proportion between the two groups. Although the CD4^+^ T‐cell proportion and the CD4^+^/CD8^+^ ratio in the recurrence group was significantly lower than that in the non‐recurrence group (Table 2).

**Table 2:** Comparison of inflammatory factors and T‐cell subpopulations between two groups of patients.

Group	Recurrent (*n* = 100)	Nonrecurrent (*n* = 100)	*t*	*p*
IL‐6 (pg/mL)	4.79 ± 1.42	3.87 ± 1.48	4.479	**.000**
TNF‐α (pg/mL)	87.99 ± 20.85	65.71 ± 5.86	10.281	**.000**
IFN‐γ (pg/mL)	1.92 ± 0.49	2.48 ± 0.87	−5.649	**.000**
CD3^+^T (%)	34.14 ± 14.33	36.86 ± 15.11	−1.306	.193
CD4^+^T (%)	7.98 ± 3.31	9.24 ± 4.29	−2.329	**.021**
CD8^+^T (%)	3.78 ± 2.12	3.83 ± 1.65	−0.195	.846
CD4^+^/CD8^+^	2.76 ± 1.42	3.81 ± 3.76	−2.624	**.009**

Abbreviations: IFN‐γ, interferon‐γ; IL‐6, interleukin‐6; TNF‐α, tumor necrosis factor‐α.

### 3.3 Logistic regression analysis of factors affecting patient recurrence

The statistically significant indicators (serum inflammatory factor levels and T‐cell subpopulation ratios) in the univariate analysis in Table 2 are assigned as independent variables. Logistic regression equation analysis suggests that serum inflammatory factor levels (IL‐6, IFN‐γ, and TNF‐α) are the influencing factors for patient recurrence (*p* < .05, Table 3).

**Table 3:** Logistics regression analysis of confounding factors affecting recurrence of epithelial ovarian cancer patients.

Parameter	*β*	Wald	*p*	OR (95% CI)
IL‐6	.404	8.472	.004	1.498 (1.141–1.196)
IFN‐γ	−1.030	12.377	.000	0.357 (0.201–0.634)
TNF‐α	.110	34.587	.000	1.116 (1.076–1.157)
CD4^+^T	.204	6.434	.011	0.816 (0.697–0.955)
CD4/CD8	−.152	2.224	.136	1.164 (0.953–1.421)

Abbreviations: IFN‐γ, interferon‐γ; IL‐6, interleukin‐6; TNF‐α, tumor necrosis factor‐α.

### 3.4 ROC curve analysis of serum inflammatory factor levels predicting ovarian cancer recurrence

**Figure 2:**
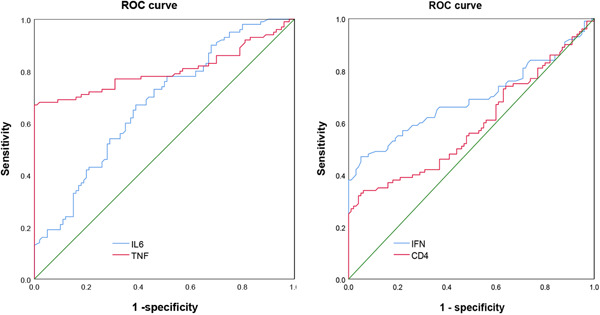
Receiver operating curve (ROC) curve of serum interleukin‐6 (IL‐6), tumor necrosis factor‐α (TNF‐α), and interferon‐γ (IFN‐γ) level predicting ovarian cancer recurrence.

Our results show that the specificity of serum TNF‐α in predicting recurrence in ovarian cancer patients and the area under the ROC curve are higher than IL‐6 and IFN‐γ (Table 4 and Figure 2).

**Table 4:** Diagnostic value of ROC curve for inflammatory factors and T‐cell subpopulations.

Parameter	AUC	Cutoff value	Sensitivity (%)	Specificity (%)	*p*	95% CI	
						Lower	Upper
IL‐6	0.674	3.54 pg/mL	49.0	78.0	.000	0.600	0.747
IFN‐γ	0.691	2.57 pg/mL	92.0	48.0	.000	0.616	0.767
TNF‐α	0.802	74.83 pg/mL	100.0	67.0	.000	0.735	0.869
CD4^+^T	0.590	12.81%	95.0	33.0	.027	0.510	0.670

Abbreviations: IFN‐γ, interferon‐γ; IL‐6, interleukin‐6; ROC, receiver operating curve; TNF‐α, tumor necrosis factor‐α.

### 3.5 Comparison of inflammatory factors and T‐lymphocyte levels between conventional therapy group and combined therapy group

**Figure 3:**
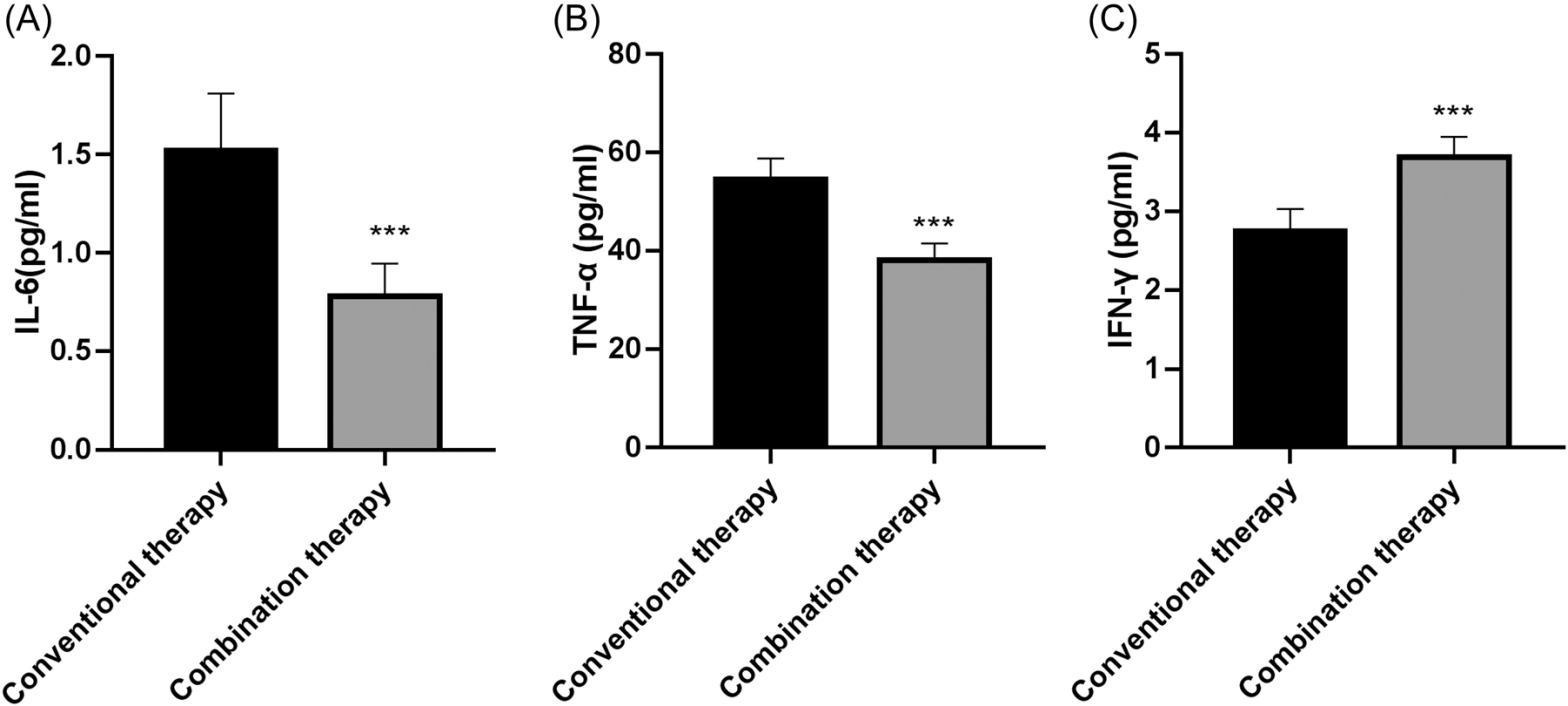
Olaparib maintenance therapy significantly inhibited the levels of interleukin‐6 (IL‐6), tumor necrosis factor‐α (TNF‐α), and interferon‐γ (IFN‐γ) in patients with recurrent ovarian cancer. The peripheral blood of the patients in conventional therapy group (*n* = 50) and conventional therapy combined with olaparib maintenance therapy group (*n* = 50) were collected, and the serum was separated. The level of IL‐6 (A), TNF‐α (B), and IFN‐γ (C) in the serum of the two groups was detected by enzyme‐linked immunosorbent assay kits. ***p < .001 versus conventional therapy group.

Based on the above results, 100 recurrent patients were divided into 2 groups according to their treatment methods: conventional therapy group (*n* = 50) and combined therapy group (*n* = 50). As shown in Figure 3A,B, compared with the conventional therapy group, the serum IL‐6 and TNF‐α level in the combined therapy group was decreased, whereas the IFN‐γ level was increased (Figure 3C). These data demonstrated that olaparib maintenance therapy significantly decreased the levels of IL‐6 and TNF‐α, but increased IFN‐γ level in patients with recurrent ovarian cancer.

**Figure 4:**
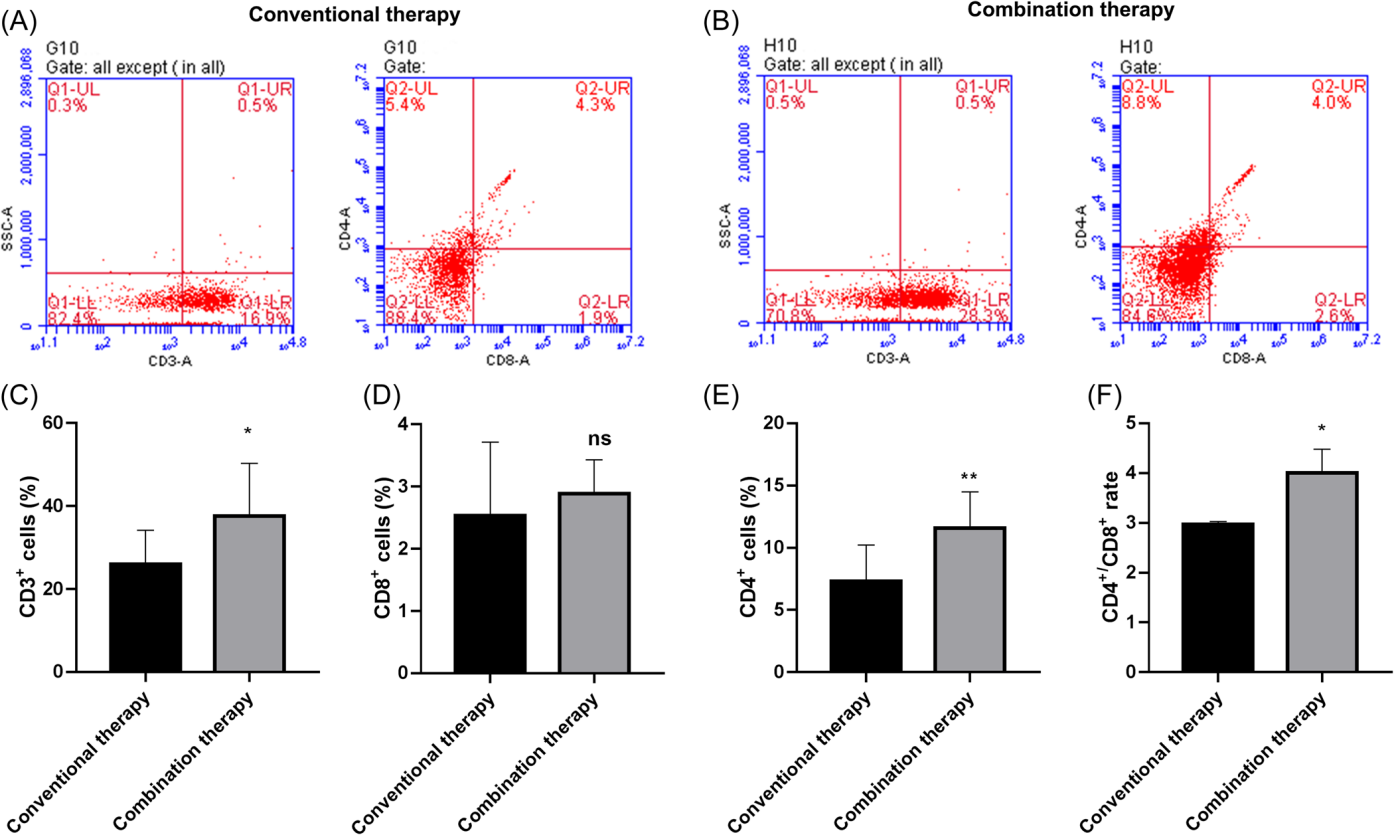
Olaparib maintenance therapy significantly increased the proportion of CD4^+^ T cells and the CD4^+^/CD8^+^ ratio in patients with recurrent ovarian cancer. The proportion of T‐cell subsets in peripheral blood of patients in conventional therapy group (*n* = 50) (A) and conventional therapy combined with olaparib maintenance therapy (*n* = 50) (B) group were detected by flow cytometry. The proportion of CD3^+^ T (C), CD8^+^ T (D), CD4^+^ T (E) cells, and CD4^+^/CD8^+^ ratio (F) in peripheral blood of patients in two groups. *p < .05, **p < .01 versus conventional therapy group.

As shown in Figure 4A,B, compared with the conventional therapy group, the peripheral blood CD3^+^ T and CD4^+^ T‐cell proportion in the combined therapy group was significantly increased (Figure 4C,E). Moreover, compared with the conventional therapy group, the CD4+/CD8+ ratio in the combined treatment group was increased (Figure 4F). These results suggested that olaparib maintenance therapy significantly increased the CD4^+^ T‐cell proportion and the CD4^+^/CD8^+^ ratio in patients with recurrent ovarian cancer.

## 4 DISCUSSION

The decrease of the CD4^+^/CD8^+^ ratio is an important feature of immune deficiency disease.^20^ The T‐lymphocyte subsets in the peripheral blood of tumor patients were abnormal, including the significant decrease of CD3^+^ and CD4^+^ T cells, the significant increase of CD8^+^ T cells, and the significant decrease of the CD4^+^/CD8^+^ ratio.^18^ These abnormal T‐lymphocyte subsets indicate that the cellular immune function of tumor patients is in an immunosuppressive state, and the ability of patients to recognize and kill mutant cells was decreased, resulting in the growth and metastasis of the tumor. In this study, the recurrent patients have the lower CD4^+^/CD8^+^ ratio, indicating the immune deficiency in recurrent patients, which may also be one of the reasons for their recurrence. But logistic regression equation analysis shows that the CD4^+^/CD8^+^ ratio are not the influencing factors for patient recurrence.

Research shows that the change of cytokine level will induce many diseases, and cytokines have the function of regulating human immune ability.^22^ IL‐6 is a cytokine that can be derived from monocytes, endothelial cells, and even glial cells, and its content is low in the normal human body.^23^ IL‐6 expression is abnormal only when an inflammatory reaction occurs or there is a tumor, and it participated in multiple cellular functions.^24^ Studies have shown that when malignant tumors progress, the function of T lymphocytes is damaged, the expression of IL‐6 can be enhanced, aggravating the inflammatory response, leading to the weakening of immune regulation function and the decline of immune level.^25^ Therefore, the expression of IL‐6 can be enhanced in the process of malignant tumor progression. Previous studies have shown that the serum IL‐6 level in ovarian cancer patients is abnormally elevated.^26^ In this study, the serum IL‐6 level in recurrence ovarian cancer patients was significantly higher than that in nonrecurrence ovarian cancer patients. Logistic regression equation analysis suggests that serum IL‐6 is the influencing factors for patient recurrence and the ROC curve shows that serum IL‐6 levels have a certain diagnostic effect (AUC = 0.674) on ovarian cancer recurrence. TNF‐α is the initiating factor of the inflammatory response, which can amplify and aggravate inflammatory response. When the body is seriously injured, serum TNF‐α level will be significantly increased. At the same time, the high TNF‐α expression can inhibit the immune function and then increase the risk of further injury.^27^ Studies have shown that the TNF‐α level is abnormally high in patients with ovarian cancer.^22^, ^28^ Here we found that recurrent patients have higher serum TNF‐α level than nonrecurrent patients. Logistic regression equation analysis suggests that serum TNF‐α is the influencing factors for patient recurrence, and the ROC curve shows that serum TNF‐α levels have a certain diagnostic effect (AUC = 0.802) on ovarian cancer recurrence.

As a characteristic cytokine, IFN‐γ can play a role in cell regulation, killing tumor cells by macrophages and regulating the immune response. Previous studies showed that the IFN‐γ level in ovarian cancer patients was lower than the normal healthy controls.^29^ Our study showed that recurrent patients have lower serum IFN‐γ level than nonrecurrent patients. Logistic regression equation analysis suggests that serum IFN‐γ is the influencing factors for patient recurrence, and the ROC curve shows that serum IFN‐γ levels have a certain diagnostic effect (AUC = 0.691) on ovarian cancer recurrence.

An international clinical trial confirmed that olapalene has great benefits for recurrent ovarian cancer patients after surgery and significantly reduces mortality.^11^ Ding et al.^30^ have reported that in mice bearing Brca1‐deficient ovarian tumors, olaparib triggers strong antitumor immunity through STING‐dependent antitumor immune response. A research reported by Sun et al.^31^ showed that olaparib through SDF1α/CXCR4 axis inhibits MDSC migration and enhances antitumor activity of CAR‐T‐cell therapy. The above research showed the regulatory effect of olaparib on the immune system. In this study, the CD4^+^/CD8^+^ ratio of patients receiving routine treatment was significantly lower than that of patients receiving olaparib maintenance treatment, indicating that olaparib maintenance treatment can improve the immune function of recurrent patients, which was consistent with previous findings. Moreover, it was reported that olaparib has a significant anti‐inflammatory effect. Such as olaparib significantly reduces the protein expression of STAT‐6 and GATA‐3, inhibits the inflammasome signaling, and reduces the remodeling characteristics associated with chronic asthma in mice.^32^ Without changing IκBα phosphorylation, olaparib can passivate the phosphorylation of P65 in Ser 536, and reduce elastase induced lung inflammation and emphysema in mice.^33^, ^34^ This study found that the serum IL‐6 and TNF‐α level of patients receiving olaparib maintenance treatment was lower than that of patients receiving conventional treatment, while the serum IFN‐γ level was increased, indicating olaparib maintenance treatment can significantly inhibit the inflammatory response of patients with ovarian cancer, which was in agreement with previous reports.

Additionally, this study lacks longitudinal monitoring of changes in the proportion of inflammatory factors and T lymphocytes in patients undergoing various chemotherapy cycles. In the future, we still need to further observation. Despite its limitations, this study can clearly demonstrate the conclusions.

In conclusion, our study found that the increased IL‐6 and TNF‐α levels in recurrent ovarian cancer patients, while the decreased IFN‐γ level, the CD4^+^ T‐cell proportion, and the CD4^+^/CD8^+^ ratio. The levels of serum IL‐6, TNF‐α, and IFN‐γ are helpful to the clinical diagnosis of ovarian cancer recurrence, and the specificity of serum TNF‐α in predicting recurrence in ovarian cancer patients and the area under the ROC curve are higher than IL‐6 and IFN‐γ.

Furthermore, olaparib maintenance therapy can significantly inhibit the IL‐6 and TNF‐α level, and increase IFN‐γ level, the proportion of CD4^+^ T cells, and the CD4^+^/CD8^+^ ratio, indicating that olaparib maintenance therapy has a significant therapeutic effect on recurrent ovarian cancer and can be used as the preferred treatment for patients with recurrent ovarian cancer.

## AUTHOR CONTRIBUTIONS

**Gulijianati Maowulieti**: Conceptualization; funding acquisition; investigation; project administration; supervision; writing—original draft; writing—review & editing. **Shaojie Zhao**: Data curation; formal analysis; investigation; methodology. **Min Zhao**: Data curation; formal analysis; investigation; methodology. **Hua Yuan**: Resources; software; validation; visualization.

## CONFLICT OF INTEREST STATEMENT

The authors declare no conflict of interest.

## Data Availability

The data that support the findings of this study are available from the corresponding author upon reasonable request.
